# Association Between Sphericity and Ventricular Function in Fetus of Diabetic Mother: A Longitudinal Study from Fetal to Neonatal Period

**DOI:** 10.1007/s00246-025-03882-w

**Published:** 2025-05-26

**Authors:** Ayman F. Sabry, Patrick D. Evers, Erin J. Madriago

**Affiliations:** 1 Pediatric Cardiology, Department of Pediatrics, Suez Canal University, Ismailia, Egypt; 2 The Doernbecher Children’s Hospital, Oregon Health & Science University, Portland, OR USA

**Keywords:** Echocardiography, Fetus, Cardiac function, Diabetes, Sphericity index

## Abstract

The impact of maternal diabetes on the fetal heart is complex and not fully understood. The study aimed to investigate how different types and stages of maternal diabetes affect fetal heart function and ventricular remodeling during prenatal and postnatal periods. The study included 45 pregnant women with diabetes and 15 with uncomplicated pregnancies. Fetal echocardiograms were performed between 28 and 32 weeks of gestation using different techniques to assess cardiac function. Measurements of ventricular transverse and longitudinal diameters, as well as the sphericity index, were determined. Postnatal follow-up echocardiograms were also performed. The diabetic pregnant group was divided into three subgroups: 15 with GDM and 30 with PGD, including 15 wellcontrolled and 15 uncontrolled cases. The E and E/A ratio values for the mitral and tricuspid valves were significantly lower in fetuses and neonates affected by diabetes compared to the healthy group. The Tei-Doppler index for the left ventricle was significantly higher in pregnancies affected by diabetes, affecting both fetuses and neonates. The right ventricle exhibited significant differences in all groups of diabetic pregnancies in fetuses and postnatally in cases of pregestational diabetes. The sphericity index differed significantly in all fetal diabetic groups and in neonates compared to controls, except for the LV in the GDM cohort. Postnatally, the sphericity index of the RV increased compared to fetal values, while the LV sphericity index remained unchanged. Maternal diabetes mellitus affects fetal cardiac function and structure, which may continue into the early days of life.

**Supplementary Information:**

The online version contains supplementary material available at 10.1007/s00246-025-03882-w.

## Introduction

Diabetes mellitus (DM) is a relatively common medical disorder of pregnancy affecting about 7% of the pregnant population and it includes pregestational diabetes (PGD) and gestational diabetes mellitus (GDM) [1, 2]. DM not only leads to maternal complications in later life of mother but also immediate and later fetal complications [2, 3]. In all the diabetic pregnancies GDM accounts for more than 80% and the rest are PGD (type I and type II) [1]. In PGD, high maternal glucose level appears before pregnancy; in contrast to GDM, abnormal maternal glucose level starts from second to third trimester [2, 3].

DM is a systemic chronic metabolic disorder characterized by increased insulin resistance and/or β-cell defects [4, 5]. Intrauterine exposure to high level of maternal blood glucose significantly increases the insulin secretion in fetal pancreas, leading to fetal hyperinsulinemia, and the fetal heart is one of the major organs affected by hyperinsulinemia [3–5]. The rapid cell proliferation and differentiation during fetal growth are very sensitive to any changes in environment leading to permanent alteration in the cardiac structural and functional constitution [5–7].

Cardiac function impairment in the fetus of the diabetic mother during the second and third trimester is well established and the timing and severity of hyperglycemia in relation to gestational age are a critical factor that determines these effects [8, 9]. The diastolic dysfunction represents the earliest manifestation of diabetic cardiomyopathy, preceding the systolic dysfunction [9, 10]. Ventricular remodeling encompasses alternation in ventricular mass, shape, and geometry as a result of an increased hemodynamic load, neurohormonal activation, and genetic factors [11–13]. The sphericity index (SI) is used to evaluate the change of the shape of the right and left ventricles [11, 12]. Less is known about changes in ventricular geometry and remodeling during fetal life among different diabetic levels and its association with cardiac dysfunction and the persistence of these changes in neonatal period.

Therefore, the purpose of this study was to provide a detailed overview of the fundamental mechanism of ventricular remodeling and its relation to cardiac function for different types and levels of DM during pregnancy and postnatally.

## Patients and Methods

This present study was designed as a prospective cohort study conducted between March 2022 and March 2023. All pregnant women referred to Suez Canal University hospital, Ismailia, Egypt for routine evaluation with diabetes mellitus. A total of 60 pregnant mothers between 28th and 32nd gestational age were examined. Forty five were in the diabetic pregnancies, and the control group was formed by fifteen healthy pregnant women matching the gestational weeks who were selected randomly from all women for routine antenatal care.

The diabetic pregnant population was classified into three groups: 15 cases in GDM and 30 PGD (15 well controlled and 15 uncontrolled), and re-evaluation was done to all newborns of our study groups after delivery. Cases were excluded if they had congenital heart disease, intrauterine growth restriction (IUGR), fetal arrhythmias, fetal structural or chromosomal abnormality, multiple pregnancy, maternal chronic disease other than diabetes mellitus and maternal hypertension.

Details of history, maternal, age, weight, parity, and gravidity were taken. Gestational ages were determined both by last menstrual periods and crown-rump length measurement on first-trimester ultrasound. Glycemic control was classified as normal if glycosylated hemoglobin (HbA1C) was < 5.7 g/al, the mean cutoff value is 6.5%, level at < 6.5% indicated wellcontrolled DM, and level > 6.5% uncontrolled DM [1, 2]. Routine obstetric ultrasound scans were noted. The diagnosis of gestational diabetes was made if one or more of the following criteria were met: fasting plasma glucose level ≥ 92 mg/dl, a one-hour level of 180 mg/dl and two-hour level ≥ 153 mg/dl following a 75 g oral glucose [2, 13]. Management of diabetic pregnancies, by insulin or metformin, when dietary management failed. The study was approved by the local ethics committee of our hospital and written consent was obtained from all study participants.

### Fetal Echocardiography

It was performed using Philips CX50 equipped with either S5-1, Convex C5-1, with standard fetal software for analysis.

All recordings, used for measurement, were obtained in the absence of fetal breathing and without maternal and fetal movement, with fetal heart rate between 120 and 160 beats per minute. Fetal 2D, pulsed-wave Doppler, and tissue Doppler imaging (TDI) were used to assess cardiac geometry and cardiac function.

The thickness of intraventricular septum was measured by averaging M-mode and 2D measurements. The 2D measurement was taken at end-diastole in a transverse four-chamber view, halfway between the apex and the heart’s crux, with a cursor perpendicular to the septum [14, 15].

Conventional diastolic function: includes mitral and tricuspid early (E) and late (A) and diastolic filling ratio (E/A ratio). Mitral and tricuspid inflow velocities were measured in apical 4-chamber view [16, 17]. Grade I diastolic dysfunction was characterized by an E/A ratio of 0.8 or lower and E velocity of 50 cm/s or less in all fetal groups. Myocardial performance index (MPI) was calculated for both right and left heart. Isovolumetric contraction time (ICT) was measured from the closure of the mitral valve to the opening of the aortic valve. Isovolumetric relaxation time (IRT) was measured from the closure of the aortic valve to the onset of the mitral opening. Ejection time (ET) is the period between aortic opening and closure. MPI = (ICT + IRT)/ET [18, 19].

RV MPI calculation: we obtained the RV inlet tract (tricuspid valve) and outlet tract curves. Measurement of “a” corresponds to the time interval between the end and beginning of RV flow which is equal to the sum of IRT + ICT + ET. Measurements of “b” = peak systolic velocity wave from the pulmonary artery obtained from the short-axis view, and it corresponds to the ET, RV-MPI = a–b/b [18, 19].

TDI was performed in the apical four-chamber view by placing a sample volume at three different sites at end-diastole: (1) Lateral border of mitral valve annulus (Left Ventricle); (2) septal wall; and (3) Lateral border of tricuspid valve annulus (Right Ventricle). The sample volume width was set as approximately 2–3 mm, and also Nyquist limit was adjusted to 15 cm/s and angle of insonation was less than 20° to avoid underestimating velocities [20–22].

The peak systolic and diastolic velocities at the LV and RV were assessed with TDI in both cases and controls. The following parameters were recorded: systolic velocity (S′), early diastolic velocity (e′), and late diastolic velocity (A′).

We calculated the ratio between early mitral inflow velocity and mitral annular early diastolic (E/e′ ratio) and the same ratio for tricuspid valve. E/e′ is an index of ventricular filling pressure. We measured the right and left E′/A′ ratio.

In the postnatal stage (assessed shortly after birth), the following were recorded: gestational maturity, birth weight, blood glucose level of newborn and all echocardiography parameters (2D, MPI, TDI, and sphericity index)were reassessed.

The left ventricular sphericity index (SI) was measured as the transverse length divided by the longitudinal length in both the apical 4 and 2 chamber views during end-diastole. The longitudinal length (LL) was measured from the apex to the mid-point of the mitral valve, and the transverse length (TL) was measured as the axis that perpendicularly intersects the mid-point of the LL. The SI was evaluated by dividing TLby LL [11, 22, 23].

Doppler evaluation including the determination of the S/D ratio and Resistance index of the umbilical artery was carried out. Resistive index (RI) = (maximum systolic velocity – end diastolic velocity /maximum systolic velocity). An abnormal RI is defined as an increased waveform index of two standard deviations above the mean of age, an absence of diastolic flow, or a reverse end diastolic flow [24, 25].

Standard fetal echocardiographic parameters were measured by single investigator (AFS). To calculate the interobserver variability, we measured the stored data of 10 randomly chosen examinations one week later (1-week interval). Three successive measurements including all of the parameters of the above were obtained and averaged.

### Statistical Analysis

Data were analyzed using student’s *T* test, and linear regression analysis was determined using the least-square method. A *p* value ≤ 0.05 was considered statistically significant. Data were presented as mean  ±  standard deviation (SD) and ranges. All statistical calculations were performed with IBM SPSS version 23.0 software. The One-way ANOVA test with post hoc analysis by least -significant difference was used .The Kruskal -Wallis test was used for comparison between more than two groups. The comparison between groups with qualitative data was done using the Chi-square test.

## Results

Sixty pregnant women were included in our study. 45 diabetic pregnancies and 15 control subjects and their neonates were included in data analysis. Diabetic pregnancies were classified according to HbA1C GDM (13 well controlled and 2 uncontrolled) and 30 PGD (15 well controlled and 15 uncontrolled).

Table 1 shows baseline characteristics in different study groups, and it was found that there was no statistically significant difference among the study groups in any of the maternal baseline characteristics except in maternal age where PGD (uncontrolled) was observed in older women of (*p* < 0.004). HbA1c was significantly higher in PGD uncontrolled group (8.50 ± 0.37). BMI  was significantly higher in all diabetic groups compared to the healthy group. The women’s gestational weeks at examination were comparable at the different groups (*p* < 0.544). The neonates of mothers with GDM and PGD (uncontrolled) were found to have a significantly higher birth weight (*p* < 0.006 and *p* < 0.001) than neonates of healthy control mothers.

**Table 1:** Demographic characteristics of study population

Parameter		Healthy group	Gestational diabetes	Pregestational diabetes		*P*-value
				Controlled DM	Uncontrolled DM	
		No. = 15	No. = 15	No. = 15	No. = 15	
Maternal age (years)	Mean ± SD	25.40 ± 3.91	27.53 ± 5.15	28.40 ± 4.50	30.33 ± 4.22	0.032
**P value**			**--**	0.071	**0.004**	
Parity	No parity	5 (33.3%)	3 (20.0%)	3 (20.0%)	7 (46.7%)	0.226
	One	4 (26.7%)	3 (20.0%)	4 (26.7%)	6 (40.0%)	
	More than one	6 (40.0%)	9 (60.0%)	8 (53.3%)	2 (13.3%)	
BMI			21.7 ± 2.6	24.1 ± 2.4	25.6 ± 2.8	0.001
**P value**				**0.001**	**0.001**	
HbA1C	Mean ± SD	5.14 ± 0.21	6.36 ± 0.57	6.20 ± 0.24	8.50 ± 0.37	0.001
**P value**			**--**	**0.001**	**0.001**	
Gestational Age (days)	Mean ± SD	30.23 ± 3.51	29.27 ± 1.62	30.12 ± 1.08	28.33 ± 1.10	0.054
Random blood sugar	Normoglycemic	15 (100.0%)	10 (66.7%)	10 (66.7%)	7 (46.7%)	0.015
	Hypoglycemic	0 (0.0%)	5 (33.3%)	5 (33.3%)	8 (53.3%)	
Birth weight (kg)	Mean ± SD	3.03 ± 0.26	3.19 ± 0.38	3.43 ± 0.38	3.65 ± 0.65	0.002
APGAR Score	7	0 (0.0%)	1 (6.7%)	0 (0.0%)	0 (0.0%)	0.062
	8	5 (33.3%)	7 (46.7%)	9 (60.0%)	9 (60.0%)	
	9	4 (26.7%)	4 (26.7%)	6 (40.0%)	6 (40.0%)	
	10	6 (40.0%)	3 (20.0%)	0 (0.0%)	0 (0.0%)	
Umbilical artery RI**	Mean ± SD	0.65 ± 0.53	0.66 ± 0.41	0.67 ± 0.21	0.53 ± 0.18	0.807

*P*-value > 0.05: Non significant (NS); *P*-value < 0.05: Significant (S); *P*-value < 0.01: highly significant (HS)

BMI (kg/m2): underweight (<18.5), normal weight (18.5–24.99), overweight (≥ 25)

**: Resistance index

RI showed that there was no statistically significant difference between different groups of our work (*p* < 0.807) and all values were within the normal range.

### Geometry Changes

**Fig. 1:**
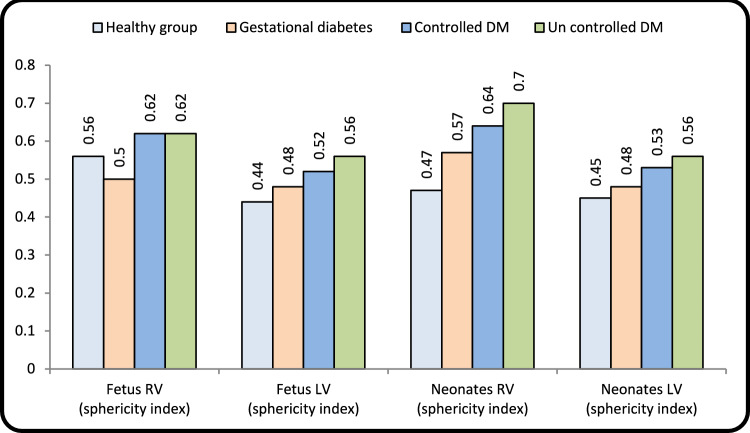
Comparison of left ventricle (LV) and right ventricle (RV) sphericity index between control and diabetic groups in fetuses and neonates

RV-GDM in fetus demonstrated decreased TL (*p* < 0.001), and LL was increased (*p* < 0.016) showing elongated shape. In neonates it changed to circular shape where TL was increased and LL was decreased.LV-GDM revealed increased LL only in fetuses and neonates. PGD showed increased TL and decreased LL in both RV and LV in fetuses and neonates presented by circular shape. Sphericity index was statistically significant in all fetal diabetic groups and in neonatal groups except for neonate LV-GDM (*p* < 0.074). Postnatal changes of diabetic mother groups as compared to fetal values revealed increased value in RV sphericity index with unchanged LV sphericity index (Table 2 and Fig. 1).

**Table 2:** Echocardiographic parameter of fetal cardiac geometry

		Healthy group	Gestational diabetes	Pregestational diabetes		*P*-value**
				Controlled DM	Uncontrolled DM	
		No. = 15	No. = 15	No. = 15	No. = 15	
Fetus						
**RV measurements**						
Longitudinal (mm)	Mean ± SD	29.17 ± 1.31	30.49 ± 1.64*	27.71 ± 1.23**	27.54 ± 1.61	0.001
	Range	26.7–31.2	27.9–33	25.8–30.2	25.1–30.2	
P-value*			**--**	**0.009**	**0.003**	
Transverse (mm)	Mean ± SD	16.26 ± 0.79	15.33 ± 0.88	17.07 ± 0.46	17.01 ± 0.63	0.001
	Range	15–17.3	13.2–16.2	16.2–17.8	15.1–17.8	
P-value*			**--**	**0.003**	**0.005**	
Fetus (RV) (sphericity index)	Mean ± SD	0.56 ± 0.04	0.50 ± 0.04**	0.62 ± 0.03**	0.62 ± 0.03**	0.001
	Range	0.5–0.64	0.45–0.58	0.57–0.68	0.56–0.67	
P-value*			**--**	**0.001**	**0.001**	
**LV measurements**						
Longitudinal (mm)	Mean ± SD	29.73 ± 1.69	27.91 ± 1.16**	26.31 ± 1.62**	26.10 ± 1.71**	0.001
	Range	27.3–33.3	25.3–29.7	23.1–28.4	24–29.3	
P-value*			**--**	**0.001**	**0.001**	
Transverse (mm)	Mean ± SD	12.99 ± 0.76	13.49 ± 0.58	13.65 ± 0.66*	14.51 ± 0.74**	0.001
	Range	11.9–14.1	12.4–14.2	12.8–14.7	13.2–15.7	
P-value*			**--**	**0.012**	**0.001**	
Fetus (LV) (sphericity index)	Mean ± SD	0.44 ± 0.03	0.48 ± 0.02**	0.52 ± 0.04**	0.56 ± 0.05**	0.001
	Range	0.39–0.49	0.44–0.52	0.46–0.60	0.45–0.64	
P-value*			**--**	**0.001**	**0.001**	
**Neonates**						
**RV measurements**						
Longitudinal (mm)	Mean ± SD	30.99 ± 1.25	29.16 ± 2.27**	26.89 ± 1.61**	26.07 ± 1.78**	0.001
	Range	29.5–33.5	25.3–33	25–31	23.9–30.1	
P-value*			**--**	**0.001**	**0.001**	
Transverse (mm)	No parity	14.41 ± 0.77	16.58 ± 1.41**	17.13 ± 0.62**	18.11 ± 0.74**	0.001
	More than one	13.1–15.5	14.1–18.5	15.2–17.8	16.7–19.4	
P-value*			**--**	**0.001**	**0.001**	
Fetus (RV) (sphericity index)	Mean ± SD	0.47 ± 0.03	0.57 ± 0.08**	0.64 ± 0.04**	0.70 ± 0.06**	0.001
	Range	0.4–0.51	0.45–0.71	0.56–0.68	0.59–0.81	
P-value*			**--**	**0.001**	**0.001**	
**LV measurements**						
Longitudinal (mm)	Mean ± SD	31.15 ± 2.19	29.36 ± 1.64*	28.61 ± 2.11**	27.28 ± 1.42**	0.001
	Range	27–35.3	26.1–32.2	25–31.5	24.3–29.5	
P-value*			**--**	**0.001**	**0.001**	
Transverse (mm)	Mean ± SD	13.97 ± 0.72	14.01 ± 0.55	15.21 ± 0.81**	15.35 ± 0.73**	0.001
	Range	12.4–15	12.4–14.6	14.2–16.7	14.1–16.7	
P-value*			**--**	**0.001**	**0.001**	
Fetus (LV) (sphericity index)	Mean ± SD	0.45 ± 0.03	0.48 ± 0.03	0.53 ± 0.06**	0.56 ± 0.04**	0.001
	Range	0.39–0.49	0.44–0.54	0.47–0.67	0.48–0.62	
P-value*			P-value*	**0.001**	**0.001**	

*P value: Compare each column with the healthy group

**P value: Compare all diabetic groups to the healthy group

RV: Right ventricle; LV: Left ventricle

### Septal Hypertrophy

There is statistically significance difference between control and PGD. In the well controlled PGD, the IVS was found in 9 fetuses and 12 neonates while in the uncontrolled PGD, it was found in 12 fetuses and 13 neonates. However, in GDM there was no statistically significant difference in fetuses and postnatally compared to the healthy group. (Appendix I and II).

### Functional Changes

#### Doppler

**Fig. 2:**
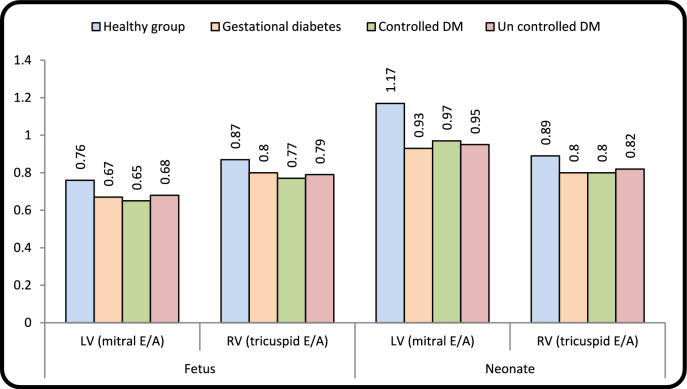
Comparison of mitral and tricuspid E/A ratio between control and diabetic groups in fetuses and neonates

The E-mitral and E-tricuspid were significantly decreased in all groups in fetuses and neonates compared to healthy group. E/A ratio for both mitral and tricuspid was statistically significant in all fetal diabetic mothers’ group and postnatal compared to control. In neonates of diabetic women, there was a significant increase (RV E/A < 1ratio and LV-E/A ≤ 1) compared to controls (Tables 3, 4, 5 and Fig. 2).

**Table 3:** Echocardiographic parameters of fetal cardiac function

Fetus		Healthy group	Gestational diabetes	Pregestational diabetes		*P*-value**
				Controlled DM	Uncontrolled DM	
		No. = 15	No. = 15	No. = 15	No. = 15	
**Mitral (Doppler)**						
LV(mitral E/A)	Mean ± SD	0.76 ± 0.08	0.67 ± 0.07**	0.65 ± 0.10**	0.68 ± 0.09*	0.005
***P*** value*		**--**	**0.006**	**0.001**	**0.011**	
Mitral E wave (cm/s)	Mean ± SD	38.87 ± 1.85	33.38 ± 3.39**	34.89 ± 3.01**	36.18 ± 1.38**	< 0.001
***P*** value*		**--**	**0.001**	**0.001**	**0.005**	
Mitral A wave (cm/s)	Mean ± SD	51.40 ± 4.24	50.16 ± 7.99	54.29 ± 6.04	55.06 ± 14.34	0.399
**Tricuspid (Doppler)**						
RV(Tricuspid E/A)	Mean ± SD	0.87 ± 0.07	0.80 ± 0.06**	0.77 ± 0.11**	0.79 ± 0.07**	0.007
***P*** value*		**--**	**0.007**	**0.001**	**0.005**	
Tricuspid E wave (cm/s)	Mean ± SD	43.89 ± 3.81	38.1 ± 2.42**	38.37 ± 3.17**	36.98 ± 2.79**	0.000
***P*** value*		**--**	**0.001**	**0.001**	**0.001**	
Tricuspid A wave (cm/s)	Mean ± SD	50.29 ± 3.77	47.70 ± 3.63	50.74 ± 6.65	47.00 ± 4.50	0.092
**Mitral (Tei Doppler)**						
ICT (sec)	Mean ± SD	23.70 ± 7.10	28.25 ± 5.00	26.60 ± 7.70	31.80 ± 5.10**	0.009
***P*** value*		**--**	0.052	0.293	**0.001**	
IRT (sec)	Mean ± SD	29.71 ± 6.50	32.15 ± 3.55	36.10 ± 3.16**	37.20 ± 3.62 **	< 0.001
***P*** value*		**--**	0.213	**0.001**	**0.001**	
ET (sec)	Mean ± SD	134.20 ± 8.70	129.02 ± 6.25	124.8 ± 3.14**	131.30 ± 8.80	0.006
***P*** value*		**--**	0.072	**0.001**	0.372	
LV MPI	Mean ± SD	0.40 ± 0.04	0.47 ± 0.03**	0.47 ± 0.02**	0.52 ± 0.05**	< 0.001
***P*** value*		**--**	**0.001**	**0.001**	**0.001**	
**Tricuspid (Tei Doppler)**						
ICT + IRT (sec)	Mean ± SD	54.70 ± 6.9	62.30 ± 6.25**	66.3 ± 12.8**	67.3 ± 6.67**	0.001
***P*** value*		**--**	**0.004**	**0.005**	**0.001**	
ET (sec)	Mean ± SD	130.20 ± 10.9	138.25 ± 12.0	141.1 ± 15.2	136.4 ± 17.1	0.194
RV MPI (sec)	Mean ± SD	0.42 ± 0.02	0.45 ± 0.05*	0.47 ± 0.04**	0.49 ± 0.03**	0.000
***P*** value*		**0.001**	**0.040**	**0.001**	**0.001**	
**Left Mitral annulus (Tissue Doppler)**						
Left annulus S’ (cm/s)	Mean ± SD	5.50 ± 0.73	5.49 ± 0.63	5.38 ± 0.68	5.35 ± 0.71	0.395
Left annulus E’ (cm/s)	Mean ± SD	7.28 ± 0.40	6.93 ± 1.09	6.43 ± 1.41*	6.23 ± 1.24*	0.048
***P*** value*		**--**	0.381	**0.038**	**0.012**	
Left annulus A’ (cm/s)	Mean ± SD	9.35 ± 1.81	7.35 ± 0.59**	8.57 ± 1.44	8.82 ± 1.83	0.005
***P*** value*		**--**	**0.001**	0.158	0.336	
E’/A’	Mean ± SD	0.80 ± 0.15	0.95 ± 0.19*	0.75 ± 0.09	0.74 ± 0.23	0.005
***P*** value*		**--**	**0.024**	0.379	0.310	
E/E’	Mean ± SD	5.36 ± 0.48	4.90 ± 0.75	5.61 ± 0.98	6.03 ± 1.25*	0.012
***P*** value*		**--**	0.175	0.448	**0.049**	
**Right Tricuspid annulus (Tissue Doppler)**						
Right annulus S’ (cm/s)	Mean ± SD	6.02 ± 0.40	5.59 ± 0.48	5.79 ± 0.43	5.82 ± 0.51	0.090
Right annulus E’ (cm/s)	Mean ± SD	7.20 ± 0.52	6.49 ± 0.46**	6.42 ± 0.43 **	6.37 ± 0.58 **	< 0.001
***P*** value*				**0.001**	**0.001**	
Right annulus A’ (cm/s)	Mean ± SD	9.46 ± 0.79	8.49 ± 1.56	9.11 ± 1.11	8.98 ± 0.97	0.152
E/E’	Mean ± SD	6.15 ± 0.59	6.39 ± 0.67	6.00 ± 0.62	5.86 ± 0.79	0.175
E’/A’	Mean ± SD	0.76 ± 0.08	0.79 ± 0.17	0.71 ± 0.09	0.72 ± 0.09	0.191

*P value: Compare each column with the healthy group

**P value: Compare all diabetic groups to the healthy group

P-value > 0.05: Non significant (NS); *P*-value < 0.05: Significant (S); *P*-value < 0.01: highly significant (HS)

^§^P < 0.05: Significant, ^§§^P < 0.01: Highly significant

**Table 4:** Echocardiographic parameters of neonate cardiac function

Neonate		Healthy group	Gestational diabetes	Pregestational diabetes		*P*-value
				Controlled DM	Uncontrolled DM	
		No. = 15	No. = 15	No. = 15	No. = 15	
**Mitral (Doppler)**						
LV (mitral E/A)	Mean ± SD	1.17 ± 0.11	0.93 ± 0.11**	0.97 ± 0.15**	0.95 ± 0.13	0.001
***P*** value*			**--**	**0.001**	**0.001**	
Mitral E wave (cm/s)	Mean ± SD	65.13 ± 1.51	58.07 ± 10.66*	59.25 ± 6.35*	56.85 ± 8.07	0.018
***P*** value*			**--**	**0.035**	**0.003**	
Mitral A wave (cm/s)	Mean ± SD	56.34 ± 7.10	63.39 ± 13.48	62.68 ± 12.31	60.98 ± 13.08	0.363
**Tricuspid (Doppler)**						
RV(Tricuspid E/A)	Mean ± SD	0.89 ± 0.04	0.80 ± 0.07**	0.80 ± 0.09**	0.82 ± 0.07	0.001
***P*** value*		**--**	**0.001**	**0.001**	**0.004**	
Tricuspid E wave (cm/s)	Mean ± SD	48.06 ± 4.20	44.10 ± 3.76*	41.83 ± 4.10**	40.13 ± 4.37	< 0.001
***P*** value*		**--**	**0.011**	**0.001**	**0.001**	
Tricuspid A wave (cm/s)	Mean ± SD	54.14 ± 4.51	55.43 ± 3.60	52.51 ± 6.02	49.29 ± 5.30	0.008
***P*** value*		**--**	0.479	0.369	**0.009**	
**Mitral (Tei Doppler)**						
ICT (sec)	Mean ± SD	20.80 ± 3.80	25.00 ± 3.15**	23.90 ± 3..20**	29.96 ± 4.10	0.001
***P*** value*		**--**	**0.003**	**0.022**	**0.001**	
IRT (sec)	Mean ± SD	22.90 ± 3.93	25.50 ± 3.70	28.27 ± 3.70**	30.70 ± 5.6	0.000
***P*** value*		**--**	0.073	**0.001**	**0.001**	
ET (sec)	Mean ± SD	121.50 ± 8.25	125.35 ± 7.22	117.80 ± 10.50	123.50 ± 9.40	0.129
***P*** value*		**--**	0.185	0.292	0.541	
LV MPI	Mean ± SD	0.36 ± 0.01	0.42 ± 0.07**	0.42 ± 0.07**	0.49 ± 0.06	0.000
***P*** value*		**--**	**0.003**	**0.003**	**0.001**	
**Tricuspid (Tei Doppler)**						
ICT + IRT (sec)	Mean ± SD	53.40 ± 3.60	55.25 ± 6.40	51.10 ± 5.10	58.50 ± 7.40	0.008
***P*** value*			**--**	0.165	**0.023**	
ET (sec)	Mean ± SD	127.31 ± 8.01	130.00 ± 7.25	124.75 ± 9.40	136.08 ± 13.6	0.018
RV MPI	Mean ± SD	0.42 ± 0.02	0.43 ± 0.03	0.41 ± 0.02	0.43 ± 0.04	0.185
***P*** value*			0.343	0.429	**0.040**	
**Left Mitral annulus (Tissue Doppler)**						
Left annulus S’ (cm/s)	Mean ± SD	5.45 ± 0.45	5.41 ± 0.34	5.26 ± 0.64	5.39 ± 0.41	0.713
Left annulus E’ (cm/s)	Mean ± SD	7.29 ± 0.77	7.06 ± 0.64	6.33 ± 0.44**	6.47 ± 0.66	0.001
***P*** value*			**--**	**0.001**	**0.001**	
Left annulus A’ (cm/s)	Mean ± SD	7.96 ± 1.18	8.01 ± 1.10	7.15 ± 0.38	7.98 ± 1.46	0.102
E’/A’	Mean ± SD	0.93 ± 0.18	0.90 ± 0.16	0.88 ± 0.04	0.84 ± 0.21	0.537
E/E’	Mean ± SD	9.03 ± 0.93	8.25 ± 1.62	9.44 ± 1.46	8.87 ± 1.62	0.164
**Right Tricuspid annulus (Tissue Doppler)**						
Right annulus S’ (cm/s)	Mean ± SD	6.18 ± 0.60	6.46 ± 0.74	6.52 ± 0.72	7.10 ± 0.27	0.001
***P*** value*			**--**	0.133	**0.001**	
Right annulus E’ (cm/s)	Mean ± SD	7.03 ± 0.27	6.64 ± 0.85	6.64 ± 0.85	5.68 ± 0.92	< 0.001
**--**			**--**	0.167	**0.001**	
Right annulus A’ (cm/s)	Mean ± SD	8.69 ± 1.09	8.68 ± 0.99	8.83 ± 1.19	8.16 ± 0.52	0.266
E/E’	Mean ± SD	6.83 ± 0.55	6.72 ± 0.85	6.37 ± 0.85	7.24 ± 1.32	0.104
E’/A’	Mean ± SD	0.82 ± 0.09	0.77 ± 0.11	0.76 ± 0.13	0.69 ± 0.08	0.018
***P*** value*			**--**	0.143	**0.002**	

*P value: Compare each column with the healthy group

**P value: Compare all diabetic groups to the healthy group

P-value > 0.05: Non significant (NS); P-value < 0.05: Significant (S); P-value < 0.01: highly significant (HS)

^§^P < 0.05: Significant, P ^§§^< 0.01: Highly significant

**Table 5:** Difference in cardiac function between fetus and neonate in different groups

		Healthy group	Gestational diabetes	Controlled DM	Uncontrolled DM	P-value
		No. = 15	No. = 15	No. = 15	No. = 15	
LV (mit E/A)	Mean ± SD	0.41 ± 0.15	0.25 ± 0.10	0.32 ± 0.21	0.27 ± 0.18	0.072
Mitral E wave (cm/s)	Mean ± SD	64.71 ± 1.50	57.6 ± 10.65**	58.77 ± 6.39**	56.34 ± 8.07**	0.004
Mitral A wave (cm/s)	Mean ± SD	4.94 ± 9.35	13.22 ± 16.48	8.40 ± 15.81	5.91 ± 21.66	0.195
RV(Tricuspid E/A)	Mean ± SD	0.01 ± 0.08	− 0.08 ± 0.06**	0.04 ± 0.06	0.03 ± 0.08	< 0.001
Tricuspid E wave (cm/s)	Mean ± SD	4.17 ± 2.64	2.90 ± 4.11	3.45 ± 3.26	3.15 ± 3.69	0.552
Tricuspid A wave (cm/s)	Mean ± SD	3.85 ± 4.51	8.15 ± 3.78*	1.77 ± 2.62	2.29 ± 5.34	< 0.001
Left annulus (S’) (cm/s)	Mean ± SD	− 0.05 ± 0.79	− 0.30 ± 0.43	− 0.12 ± 1.10	0.04 ± 0.63	0.629
Left annulus (E’) (cm/s)	Mean ± SD	0.12 ± 1.02	0.64 ± 0.72	− 0.16 ± 0.71	0.10 ± 0.90	0.062
Left annulus (A’) (cm/s)	Mean ± SD	− 1.39 ± 2.13	0.65 ± 1.30**	− 1.42 ± 1.57	− 0.84 ± 1.66	0.008
Left annulus E’/A’	Mean ± SD	0.18 ± 0.21	− 0.10 ± 0.22**	0.15 ± 0.11	0.11 ± 0.26	0.001
Left annulus E/E’	Mean ± SD	3.91 ± 1.27	2.89 ± 1.75	3.74 ± 2.10	3.24 ± 2.00	0.357
Right annulus (S’) (cm/s)	Mean ± SD	0.16 ± 0.70	0.87 ± 0.55**	0.73 ± 0.64*	1.28 ± 0.53**	0.001
Right annulus (E’) (cm/s)	Mean ± SD	− 0.13 ± 0.63	0.15 ± 1.01*	0.22 ± 0.84	− 0.69 ± 0.88	0.027
Right annulus (A’) (cm/s)	Mean ± SD	− 0.77 ± 1.35	0.18 ± 2.00	− 0.28 ± 1.59	− 0.82 ± 0.86	0.441
Right annulus E’/A’	Mean ± SD	− 5.33 ± 0.61	− 5.62 ± 0.66	− 5.24 ± 0.62	− 5.16 ± 0.81	0.309
Right annulus E/E’	Mean ± SD	6.07 ± 0.52	5.93 ± 0.89	5.66 ± 0.87	6.52 ± 1.32	0.117
(RV) Longitudinal (mm)	Mean ± SD	1.83 ± 1.28	− 1.33 ± 0.95**	− 0.82 ± 2.10**	− 1.47 ± 1.56**	< 0.001
(RV) Transverse (mm)	Mean ± SD	− 1.85 ± 0.82	1.25 ± 1.28**	0.06 ± 0.79**	1.09 ± 0.84**	< 0.001
(RV) Sphericity index	Mean ± SD	− 0.09 ± 0.03	0.07 ± 0.05**	0.02 ± 0.06**	0.08 ± 0.06**	< 0.001
(LV) Longitudinal (mm)	Mean ± SD	1.43 ± 1.70	1.45 ± 1.40	2.30 ± 2.03	1.18 ± 2.13	0.481
(LV) Transverse (mm)	Mean ± SD	0.97 ± 0.65	0.52 ± 0.55	1.57 ± 0.96	0.83 ± 0.56	0.008
(LV) Sphericity index	Mean ± SD	0.01 ± 0.04	− 0.01 ± 0.03	0.01 ± 0.05	0.01 ± 0.05	0.412

*P*-value > 0.05: Non significant (NS); *P-value < 0.05: Significant (S); **P-value < 0.01: highly significant (HS)

‡: Kruskal Wallis test was used, *P < 0.05: Significant, **P < 0.01: Highly significant

#### Tei-Doppler Changes

**Fig. 3:**
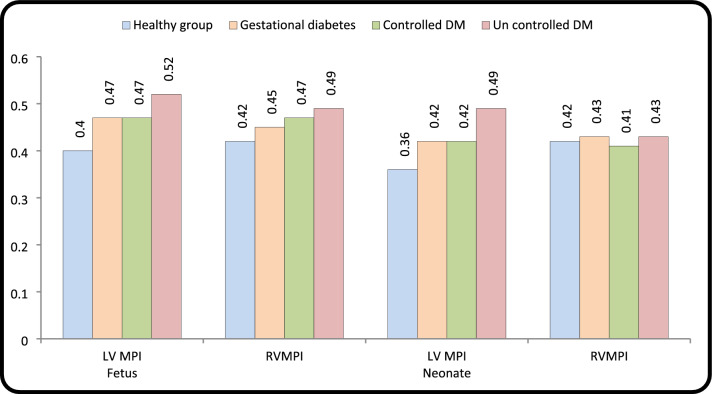
Comparison of left ventricle MPI and right ventricle (RV) MPI between control and diabetic groups in fetuses and neonates

MPI-LV was significantly higher in diabetic pregnancies than in control for both fetuses and neonates. MPI-RV was statistically significant in all groups of diabetic pregnancies in fetuses. PGDLV-IRT was statistically significant compared to control for both fetuses and neonates. LV-IRT for GDM showed (p, 0.213 and *p* < 0.073) in fetus and neonate respectively. RV-ICT + IRT was significantly prolonged in fetuses of all diabetic pregnancies and in PGD (uncontrolled) in neonate. Postnatal changes in the diabetic group demonstrated a significant decrease in LV-MPI and in PGD- RV-MPI as both RV and LV relaxation time decreased significantly in neonates compared to fetuses in the diabetic group (Tables 3, 4, 5, and Fig. 3).

#### Tissue Doppler

The S’ wave velocities of the left ventricle and right ventricle (RV) were lower but not statistically significant in the cases compared to the control group. RV demonstrates evidence of diastolic dysfunction in all groups of diabetic pregnancies as they have lower E′ wave velocity. Diastolic dysfunction was presented in LV-PGD as E ‘(left annulus) velocity was significantly lower in fetus of PGD (well controlled) and (uncontrolled) (*p* < 0.038 and *p* < 0.012), respectively, E (left annulus) in GDM was (*p* < 0.381). E/e′ was statistically significant in fetuses of PGD (uncontrolled) *p* < 0.049.

Postnatal E′ (left annulus) was significant in PGD and E′ (right annulus) was statistically lower in PGD (uncontrolled) (*p* < 0.001) (Tables 3, 4, and 5).

## Discussion

This study is the first to comprehensively evaluate the fetal cardiac remodeling, structure and function under different maternal glucose levels of diabetic mothers and postnatal follow-up. The study indicates that fetuses of diabetic women show notable alterations in cardiac structure,myocardial function and ventricular performance, which could endure postnatally.Ventricular remodeling encompasses alternation in ventricular mass, shape and geometry as a result of stressful stimuli [26–28]. The pathophysiology behind the effect of maternal diabetes on the fetal heart is complex and multifactorial and is still incompletely understood [5, 11, 29, 30]. Umbilical artery RI studies demonstrated that placental circulation remains essentially normal throughout cardiac pregnancies. Nizard reported that diabetic pregnancy is not associated with a significantly higher incidence of abnormal umbilical artery RI on Doppler study than non-diabetic pregnancy [24, 25]. We could not sign out pressure or volume overload as a pathogenesis for these changes, but these changes may be related to intrinsic myocardial ones.

Cardiac function and structural changes may be related to the fetal hyperinsulinemia, or reactive oxygen originating from oxidative stress circulating in diabetic maternal blood [3, 5, 6]. This could directly disturb the structure and function of fetal heart in the form of myocardial changes such as inflammation, mitochondrial dysfunction, neurohumoral activation, myocyte hypertrophy, decreased myocyte viability and fibroblast proliferation [3, 6, 30]. Hyperglycemia in GDM on temporary peaks, during the third trimester of pregnancy is essential for the development of myocardial hypertrophy and diastolic heart abnormality [4, 30, 31]. These findings are found earlier in the babies of mothers with pregestational diabetes and later in those with gestational diabetes.

### Cardiac Sphericity and Remodeling

Our study demonstrated that early geometrical changes in fetal heart as detected in GDM were elongated RV (increase in longitudinal diameter and decrease in transverse diameter significantly) and followed by affection of LV (by increase in longitudinal diameter). It is explained by stress power distributed equally on both sided of RV, while increase in longitudinal direction leads to change from ellipsoid (normal ventricle) to elongated shape [11, 12, 32].

**Fig. 4:**
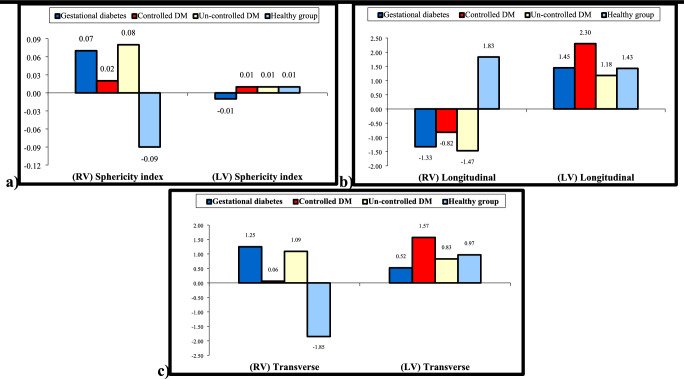
**a** Changes in the mean sphericity index of the left and right ventricles between fetuses and neonates in each group **b** Changes in the mean longitudinal diameter of the left and right ventricles between fetuses and neonates in each group. **c** Changes in the mean transverse diameter of the left and right ventricles between fetuses and neonates in each group. P.S. The negative value indicates reduction after delivery and positive value indicate increase after delivery

**Fig. 5:**
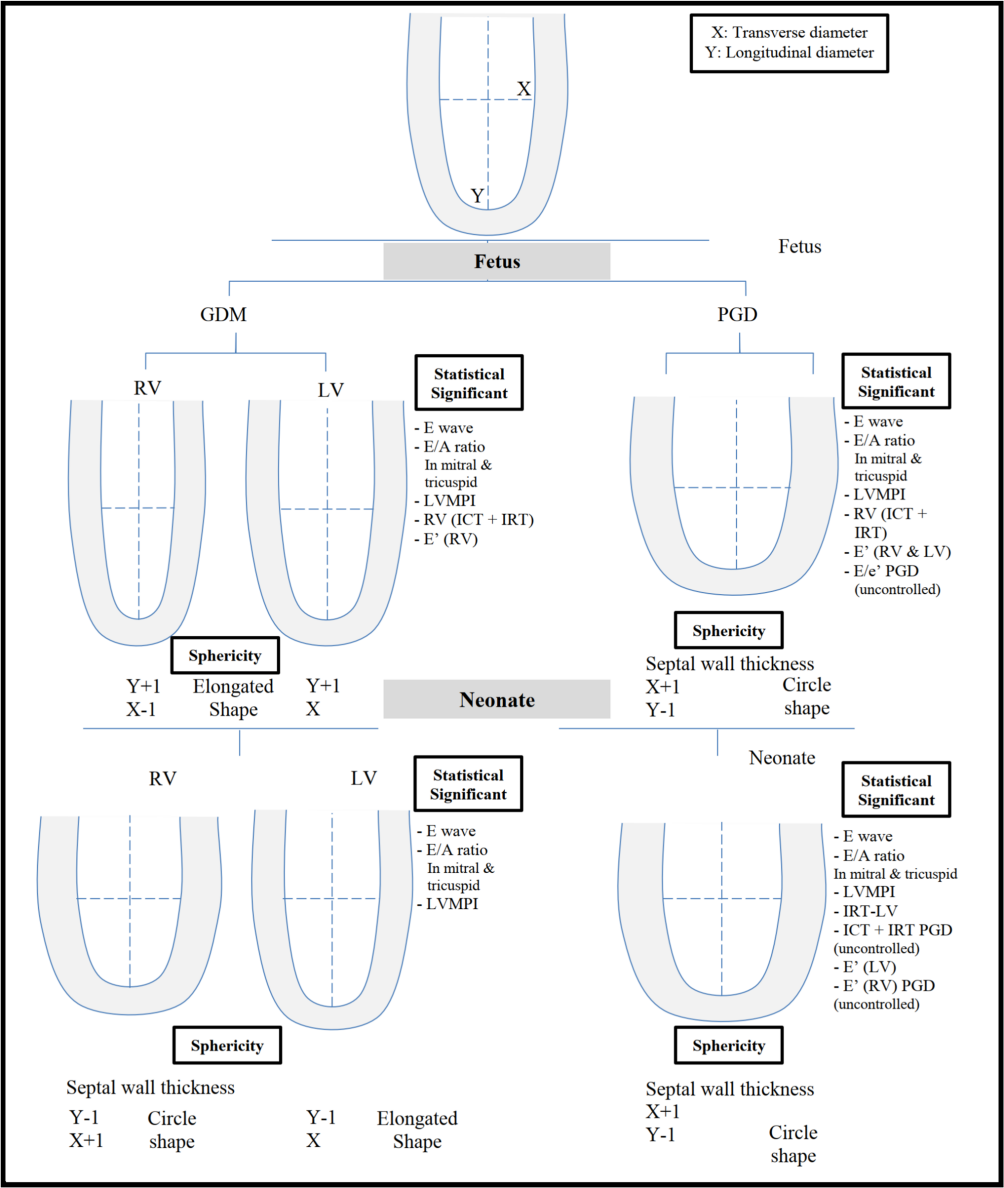
Diagram summarizing inclusion in systemic review of the study on effect of maternal diabetes on fetal and neonatal cardiac function and geometry

We found circular shape in PGD group in fetuses and in all diabetic groups postnatally. This is due to wall stress being more in transverse diameter and decreased in longitudinal diameter as myocytes can still function appropriately without the need for increased force development (hypertrophy) and as the continuous action of horizontal stress force component produces septal hypertrophy [13, 26, 33]. This is done in order to generate higher force and pressure with lower stress on the individual myocytes and less energy consumption [13, 26], Postnatally, all diabetic groups exhibited persistence of significant increase of LV and RV sphericity indices. These changes into circular shape for both LV and RV postnatally proved by the change of the shape of the ventricle starting elongated then changed into circular shape later on [11, 12, 28]. Our study showed that fetuses and neonates of diabetic women demonstrated significantly increased RV and LV sphericity index compared to healthy control. Our data demonstrated that cardiac remodeling that developed in response to an adverse intrauterine environment could not regress when the insult disappears postnatally. It could be explained by the “fetal programming hypothesis” which causes structural changes and persists postnatally [34]. Increase RV afterload from elevated pulmonary hypertension. could be another reason for persistence of RV sphericity shape postnatally [10] (Figs. 4 and 5). Changes in cardiac structure and shape are often accompained by subclinical alterations in the heart's function leading to cardiac dysfunction.

### Septal Wall Thickness

Septal wall thickness was statistically significant in PGD groups for fetuses and neonates and only in two fetuses and one neonate in GDM with no statistical significance. We did not find any differences of IVS thickness between uncontrolled and wellcontrolled PGD. Depla et al. showed IVS thickness had significantly higher values in PGD pregnancies than in control group [33, 34]. Postnatal evaluation in neonates of diabetic mothers revealed cardiac hypertrophy to be present in 25–40% of neonates [11, 35–37]. Septal hypertrophy may be related to continuous action of the horizontal stress force component [13, 26, 27]. Our results suggested that early hyperglycemia presence during pregnancy had an obvious influence on IVS as in PGD [31, 36]. Spontaneous regression of hypertrophy usually resolves with normalization of plasma insulin level [5] (Appendix I and II).

### Diastolic Dysfunction (Doppler Echocardiography)

GDM revealed that remodeling starts first in RV followed by LV, and these ventricular geometry changes followed by diastolic dysfunction and later develop septal hypertrophy. This dysfunction may be related to the stiffer RV and LV myocardium in fetuses of diabetic women that might be responsible for a limited preload. This can be concluded in our study as E wave showing significant decrease in both mitral and tricuspid valves in GDM with PGD groups in fetuses and neonates [8, 16, 35]. Reduced E/A of RV and LV in fetuses of diabetic mothers group demonstrated the presence of impaired LV and RV relaxation (E/A ratio ≤ 1)) [16, 17, 21], suggesting atrial contribution to the majority of blood in diastole due to reduced ventricular compliance and diastolic dysfunction is considered grade I as E/A less than 0.8m/s and E wave less than 50 cm/s. It was revealed that E/A ratio in third trimester was lower in PGD pregnancies than in control group but not in GDM [35]. We observed that altered filling patterns of mitral and tricuspid inflow Doppler have been noted even without septal hypertrophy. We observed that E/A ratio in healthy control group was < 1 compared to the usual post-natal E/A ratio > 1. This could be explained that our results demonstrated that the E wave was initially of lower velocity due to reduced compliance of the fetal myocardium but increases throughout gestation to become more than the A wave velocity postnatally [10].

### Cardiac Function (Tei-Doppler Index)

LV-MPI was positive in all fetal diabetic groups and neonates compared to healthy control group. RV MPI was significant in fetal GDM and PGD. The main parameters of MPI-LV were increased as a result of prolonged LV-IRT [18, 20]. This work revealed that LV-IRT was significantly prolonged in PGD group and not GDM in fetuses and neonates and RV-IRT+ICT was significantly prolonged in all diabetic fetuses. Postnatally [12, 18, 21] there was reduction in RV-IRT+ICT except in PGD (uncontrolled) where RV-IRT+ICT still prolonged significantly in neonates. We suggest that relatively low value of LV Tei index in neonates compared to fetuses of diabetic groups from multiple circulatory adaptation (as increase in preload on the LV caused by ductus arteriosus and increase in oxygen consumption) led to potential elevation of myocardial contractility [18, 21] (Figs. 4 and 5).

### Cardiac Function (Tissue Doppler)

The study done by Nagueh et al. found a significant decrease in the left ventricular E′, A′, and S′ velocities in patients with hypertrophic cardiomyopathy [37–39]. In our study, the left ventricular and right ventricular S’ velocities and right ventricular A′ were found to be lower in the fetuses and as compared to the control group but not statistically significant. In our study, S wave was ≥ 5.4 cm/s in GDM and in neonates of all diabetic groups. This finding indicated that systolic functions were preserved in these groups. [38, 39]. The variable E′ is considered to indicate ventricular relaxation independent of volume load and reported diastolic dysfunction [37–39].

Tissue Doppler RV E′ wave was significantly decreased in all fetal groups compared to control group and statistically significant in PGD (uncontrolled) postnatally. On Tissue Doppler, early findings of diastolic dysfunction were significantly reduced in E′. LV E′ was significant reduced in PGD in fetuses and neonates but not in GDM [8, 18]. We also found that the velocities of the E′ of the right and left ventricular wall were lower than those of the A′ wave. This may be related to mild diastolic dysfunction. There was no difference in LV and RV E/e ratio between the control group and diabetic groups except in fetal PGD (uncontrolled) which was significant [35, 36]. These findings agree with Balli who found the same results as E/e is usually elevated in diastolic dysfunction in grade II where E wave tends to become larger and e remain low [20]. E/e was statistically significant in PGD (uncontrolled), and we can predict LV filling pressure will increase as explained by ratio α PLA (E α PLA/t P = pressure, t = time & e′ α 1/t) and as E increases significantly [11, 17, 20]. Our results demonstrated that diastolic dysfunction diagnosed with abnormal Tricuspid & Mitral E wave, E/A ratio, prolonged IRT and E′ wave and comparisons between controlled and uncontrolled PGD indicated that good maternal glycemic control might early improve fetal cardiac function impairments. This work demonstrated that RV-fetus showed more evidence of diastolic dysfunction as it has lower E velocity, E/A ratio, prolonged IRT, and higher e′ velocity.

## Clinical Perspectives

As persistence of ventricular remodeling and diastolic dysfunction, irrespective of the diabetes being pregestational or gestational postnatally, it will be a great interest to assess the long-term outcome in childhood period. Recognition of early and subtle changes may help in management and improve outcomes.

## Conclusions

In conclusion, fetuses of diabetic mothers exhibit earlier altered ventricular geometry then followed by diastolic dysfunction (grade I) and these changes present in neonatal period. This dysfunction occurred with or without septal hypertrophy and improvement in some of these cardiac indices postnatally is related to the type of DM, level of HBA1c and the degree and duration of hyperglycemia. Monitoring cardiac function and morphology is required in pregestational diabetes before and after birth, and in gestational diabetes after birth.

## Limitations

The limitation of our study was the small number of diabetic pregnancies (GDM and PGD) in our university. We did not utilize spectral tissue Doppler, a more accurate method for detecting ventricular dysfunction and automated of the fetal right myocardial performance index to improve results [40–42]. Our study group was cross sectional and limited to the second and third trimesters of gestation.

## Electronic supplementary material

Below is the link to the electronic supplementary material.

Supplementary file1 (DOCX 1229 KB)

## Data Availability

No datasets were generated or analysed during the current study.
