# Supplementary material for: Association Between Sphericity and Ventricular Function in Fetus of Diabetic Mother: A Longitudinal Study from Fetal to Neonatal Period
Source: Pediatr Cardiol. 2025 May 26;47(3):1093–108. doi: 10.1007/s00246-025-03882-w (PMC12901260; doi:10.1007/s00246-025-03882-w)
Supplement: Supplementary file 1 — Supplementary file1 (DOCX 1229 KB) [file 246_2025_3882_MOESM1_ESM.docx]

**Appendix**

**Appendix Legend**

**Appendix I:** Neonatal Septal Thickness measurement

**Appendix II:** Fetal Septal Thickness measurement

**Appendix III:** Assessment of Sphericity index

**Appendix I**

Neonatal Septal Thickness measurement

| \|  \| \| **Healthy group** \| **Gestational diabetes** \| **Controlled DM** \| **Un- controlled** \| **P-value** \| \| --- \| --- \| --- \| --- \| --- \| --- \| --- \| \| **No.= 15** \| **No.= 15** \| **No.= 15** \| **No.= 15** \| \| Neonates Sepetal thickness \| Mean ± SD \| 3.63 ± 0.29 \| 3.99 ± 0.4 \| 4.77 ± 0.68 \| 5.67 ± 0.55 \| 0.009• \| \| Range \| 3.3 – 4.3 \| 3.6 – 5.1 \| 3.7 – 5.6 \| 4.5 – 6.7 \| \| Zscore: Neonates Sepetal thickness \| Median (IQR) \| -0.99 (-1.2 - -0.88) \| -0.77 (-0.88 - -0.23) \| 0.2 (-0.34 - 0.95) \| 1.28 (0.95 - 1.6) \| 0.002≠ \| \| Range \| -1.31 – -0.23 \| -0.99 – 0.63 \| -0.88 – 1.17 \| -0.02 – 2.35 \| \| Sepetal thickness groups \| (3-4) ml \| 13 (86.7%) \| 10 (66.7%) \| 3 (20%) \| 0 (0%) \| 0.000* \| \| (4-5) ml \| 2 (13.3%) \| 4 (26.7%) \| 5 (33.3%) \| 2 (13.3%) \| \| (5 - 6)ml \| 0 (0%) \| 1 (6.7%) \| 7 (46.7%) \| 9 (60%) \| \| (6 -7) ml \| 0 (0%) \| 0 (0%) \| 0 (0%) \| 4 (26.7%) \|   P-value > 0.05: Non significant; P-value < 0.05: Significant; P-value < 0.01: Highly significant  *: Chi-square test; •: One Way ANOVA test; ≠: Kruskal-Wallis test |  |
| --- | --- | --- | --- | --- | --- | --- | --- | --- | --- | --- | --- | --- | --- | --- | --- | --- | --- | --- | --- | --- | --- | --- | --- | --- | --- | --- | --- | --- | --- | --- | --- | --- | --- | --- | --- | --- | --- | --- | --- | --- | --- | --- | --- | --- | --- | --- | --- | --- | --- | --- | --- | --- | --- | --- | --- | --- | --- | --- |
| 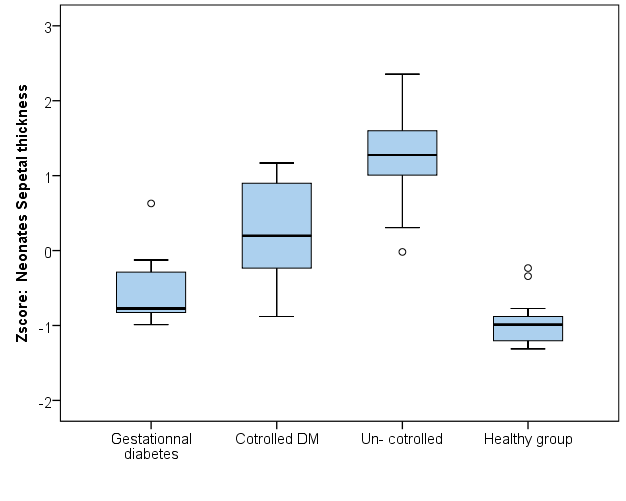 |  |

**Appendix II**

Fetal Septal Thickness measurement

| \|  \| \| **Healthy group** \| **Gestational diabetes** \| **Controlled DM** \| **Un- controlled** \| **P-value** \| \| --- \| --- \| --- \| --- \| --- \| --- \| --- \| \| **No.= 15** \| **No.= 15** \| **No.= 15** \| **No.= 15** \| \| Fetus Sepetal thickness \| Mean ± SD \| 3.55 ± 0.12 \| 4.11 ± 0.57 \| 4.89 ± 0.87 \| 5.5 ± 0.54 \| 0.001• \| \| Range \| 3.4 – 3.8 \| 3.6 – 5.5 \| 3.7 – 6.1 \| 4.6 – 6.3 \| \| Zscore: Fetus Sepetal thickness \| Median (IQR) \| -1.07 (-1.07 - -0.97) \| -0.65 (-0.86 - -0.12) \| 0.73 (-0.75 - 1.26) \| 1.04 (0.73 - 1.57) \| 0.000≠ \| \| Range \| -1.18 – -0.75 \| -0.97 – 1.04 \| -0.86 – 1.68 \| 0.09 – 1.89 \| \| Sepetal thickness \| (3-4) ml \| 15 (100%) \| 10 (66.7%) \| 5 (33.3%) \| 0 (0%) \| 0.000* \| \| (4-5) ml \| 0 (0%) \| 3 (20%) \| 1 (6.7%) \| 3 (20%) \| \| (5 - 6)ml \| 0 (0%) \| 2 (13.3%) \| 8 (53.3%) \| 8 (53.3%) \| \| (6 -7) ml \| 0 (0%) \| 0 (0%) \| 1 (6.7%) \| 4 (26.7%) \|   P-value > 0.05: Non significant; P-value < 0.05: Significant; P-value < 0.01: Highly significant  *: Chi-square test; •: One Way ANOVA test; ≠: Kruskal-Wallis test |  |
| --- | --- | --- | --- | --- | --- | --- | --- | --- | --- | --- | --- | --- | --- | --- | --- | --- | --- | --- | --- | --- | --- | --- | --- | --- | --- | --- | --- | --- | --- | --- | --- | --- | --- | --- | --- | --- | --- | --- | --- | --- | --- | --- | --- | --- | --- | --- | --- | --- | --- | --- | --- | --- | --- | --- | --- | --- | --- | --- |
| 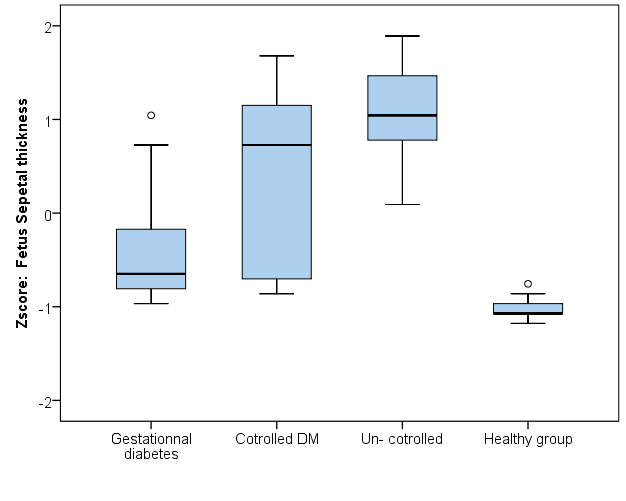 |  |

**Appendix III**

Assessment of Sphericity index


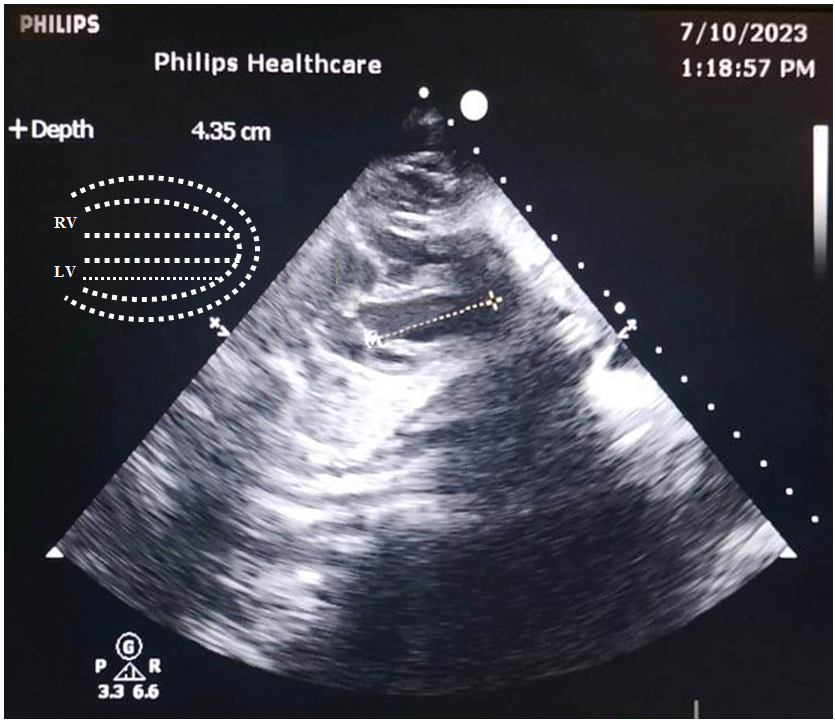

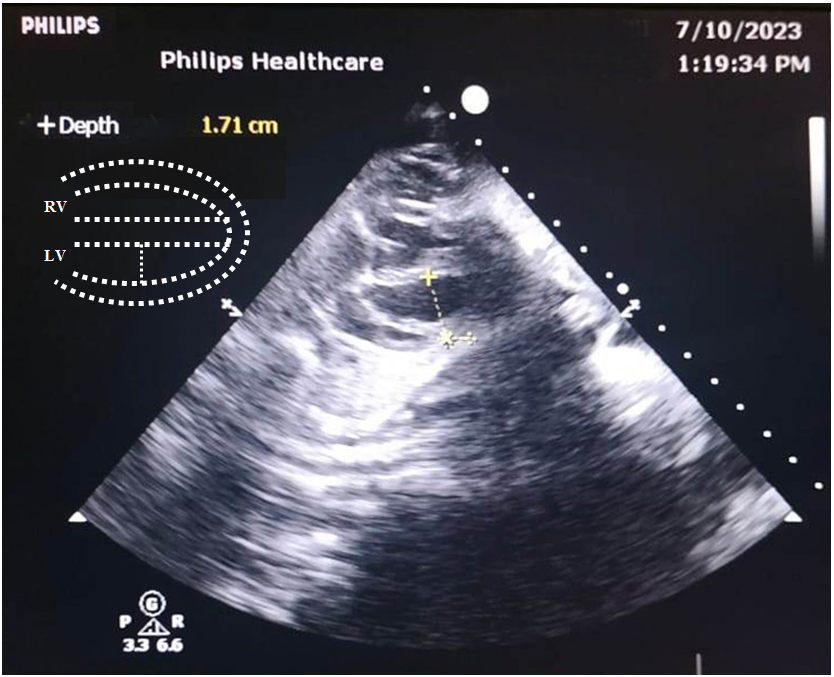


| Longitudinal diameter | Transverse diameter |
| --- | --- |

Sphericity index= transverse diameter / longitudinal diameter
